# Supplementary material for: Time-series transcriptome analysis identified differentially expressed genes in broiler chicken infected with mixed Eimeria species
Source: Front Genet. 2022 Aug 8;13:886781. doi: 10.3389/fgene.2022.886781 (PMC9393255; doi:10.3389/fgene.2022.886781)
Supplement: Supplementary file 2 [file DataSheet1.ZIP › 4dpi_KEGG.Gsea.1625100298584/KEGG_INTESTINAL_IMMUNE_NETWORK_FOR_IGA_PRODUCTION.html]

Details for gene set KEGG\_INTESTINAL\_IMMUNE\_NETWORK\_FOR\_IGA\_PRODUCTION[GSEA]

|  || Dataset | TMM\_4dpi\_gct\_format\_4dpi\_gct\_format.Class\_4dpi.cls #PC\_versus\_NC.Class\_4dpi.cls #PC\_versus\_NC\_repos |
| Phenotype | Class\_4dpi.cls#PC\_versus\_NC\_repos |
| Upregulated in class | 0 |
| GeneSet | KEGG\_INTESTINAL\_IMMUNE\_NETWORK\_FOR\_IGA\_PRODUCTION |
| Enrichment Score (ES) | -0.54876256 |
| Normalized Enrichment Score (NES) | -1.611906 |
| Nominal p-value | 0.02087961 |
| FDR q-value | 0.15902631 |
| FWER p-Value | 0.6798 |
Table: GSEA Results Summary

  

Fig 1: Enrichment plot: KEGG\_INTESTINAL\_IMMUNE\_NETWORK\_FOR\_IGA\_PRODUCTION      
 Profile of the Running ES Score & Positions of GeneSet Members on the Rank Ordered List

  

| SYMBOL | TITLE | RANK IN GENE LIST | RANK METRIC SCORE | RUNNING ES | CORE ENRICHMENT || 1 | IL15RA | na | 677 | 0.836 | 0.0287 | No |
| 2 | CCL28 | na | 1625 | 0.489 | -0.0004 | No |
| 3 | IL15 | na | 2691 | 0.325 | -0.0561 | No |
| 4 | CD80 | na | 4648 | 0.111 | -0.2080 | No |
| 5 | CD40LG | na | 8222 | -0.200 | -0.4855 | No |
| 6 | CD86 | na | 8523 | -0.232 | -0.4869 | No |
| 7 | TNFSF13B | na | 9266 | -0.317 | -0.5165 | Yes |
| 8 | CXCL12 | na | 9452 | -0.342 | -0.4971 | Yes |
| 9 | MAP3K14 | na | 9607 | -0.363 | -0.4729 | Yes |
| 10 | ICOS | na | 9720 | -0.376 | -0.4439 | Yes |
| 11 | TGFB1 | na | 9972 | -0.409 | -0.4231 | Yes |
| 12 | CD28 | na | 10233 | -0.450 | -0.3989 | Yes |
| 13 | CD40 | na | 10769 | -0.552 | -0.3872 | Yes |
| 14 | ITGA4 | na | 10879 | -0.577 | -0.3375 | Yes |
| 15 | PIGR | na | 10979 | -0.602 | -0.2844 | Yes |
| 16 | ICOSLG | na | 11035 | -0.617 | -0.2260 | Yes |
| 17 | CXCR4 | na | 11042 | -0.619 | -0.1634 | Yes |
| 18 | AICDA | na | 11146 | -0.648 | -0.1060 | Yes |
| 19 | TNFRSF13B | na | 11506 | -0.811 | -0.0532 | Yes |
| 20 | MADCAM1 | na | 11667 | -0.933 | 0.0286 | Yes |
Table: GSEA details [plain text format]

  

Fig 2: KEGG\_INTESTINAL\_IMMUNE\_NETWORK\_FOR\_IGA\_PRODUCTION      
 Blue-Pink O' Gram in the Space of the Analyzed GeneSet

  

Fig 3: KEGG\_INTESTINAL\_IMMUNE\_NETWORK\_FOR\_IGA\_PRODUCTION: Random ES distribution      
 Gene set null distribution of ES for **KEGG\_INTESTINAL\_IMMUNE\_NETWORK\_FOR\_IGA\_PRODUCTION**
